# Protein Inhibitor of Activated STAT Y (PIASy) Regulates Insulin Secretion by Interacting with LIM Homeodomain Transcription Factor Isl1

**DOI:** 10.1038/srep39308

**Published:** 2016-12-21

**Authors:** Chengzhi Yan, Chulin Yu, Di Zhang, Yan Cui, Jinlian Zhou, Sheng Cui

**Affiliations:** 1 State Key Laboratory of Agrobiotechnology, College of Biological Sciences, China Agricultural University, Beijing, China; 2 Department of Pathology and Experimental Medicine, 306 Hospital of PLA, Beijing, China

## Abstract

It is known that the LIM homeodomain transcription factor Isl1 is highly expressed in all pancreatic endocrine cells and functions in regulating pancreatic development and insulin secretion. The Isl1 mutation has been found to be associated with type 2 diabetes, but the mechanism responsible for Isl1 regulation of insulin synthesis and secretion still needs to be elucidated. In the present study, the protein inhibitor of activated STAT Y (PIASy) was identified as a novel Isl1-interacting protein with a yeast two-hybrid system, and its interaction with Isl1 was further confirmed by a co-immunoprecipitation experiment. PIASy and Isl1 colocalize in human and mouse pancreas and NIT beta cells. Furthermore, PIASy and Isl1 upregulate insulin gene expression and insulin secretion in a dose-dependent manner by activating the insulin promoter. PIASy and Isl1 mRNA expression levels were also increased in type 2 diabetic *db/db* mice. In addition, our results demonstrate that PIASy and Isl1 cooperate to activate the insulin promoter through the Isl1 homeodomain and PIASy ring domain. These data suggest that that PIASy regulates insulin synthesis and secretion by interacting with Isl1 and provide new insight into insulin regulation, although the detailed molecular mechanism needs to be clarified in future studies.

Isl1 is an important LIM homeodomain transcription (LIM-HD) factor regulating a subset of target genes in cooperation with other transcription factors^1^. Isl1 was first identified as a protein binding to the insulin gene enhancer HD consensus sequence^2^. It consists of two N-terminal LIM domains responsible for protein interaction, a central homeodomain (HD) that binds to the specific DNA sequence and a C-terminal transactivation domain^3^. Isl1 expression has been reported in motor neurons^4^, the heart^5^, the digestive system^6^, the pituitary gland^7^ and the pancreas^8^, and Isl1 is regarded as a major transcriptional regulator in the control of pattern formation and cell specification in these tissues^9^.

Insulin produced by the pancreatic islets is essential for glucose metabolism and abnormalities in insulin synthesis and secretion are linked to many diseases, including maturity-onset diabetes of the young (MODY) and type 2 diabetes^10^. Isl1 also plays important roles in the maturation and proliferation of islet cells. In Isl1 mutant embryos, dorsal pancreatic mesenchyme did not form and exocrine cell differentiation failed^11^,^12^. Isl1 also promotes adult pancreatic islet cell proliferation by activating c-Myc and CyclinD1 transcription and directly binding to their promoters^13^. Our recent studies have shown that Isl1 is involved in regulating insulin synthesis in pancreatic β cells. These findings demonstrate that Isl1 is a candidate gene for type 2 diabetes. In 1993, 3 Isl1 nonsense mutations in human beings were first screened in 75 French Caucasian patients with type 2 diabetes^14^. Another Isl1 gene mutation (Q310X) was found in a Japanese patient with type 2 diabetes, and the activity of the Q310X mutation showed a 50% reduction relative to the wild-type protein^15^. Isl1 gene mutations may be a cause of diabetes. However, the molecular mechanism underlying the interaction between Isl1 and other cofactors is not fully understood.

Protein inhibitors of activated STAT (PIAS) family proteins were originally found through their interaction with the signal transducer and activator of transcription (STAT) family of transcription factors^16^. The PIAS family consists of five members, PIAS1, PIAS3, PIASxa, PIASxb and PIASy, all of which contain several highly conserved regions including a PINIT motif involved in nuclear retention, a strikingly conserved zinc ring finger domain, a C-terminal acidic domain and a SAP domain that mediates interactions between nuclear receptors and their coregulators^17^. PIASy is an inhibitor of STAT1, and it has the shortest and least conserved C-terminal region. PIASy plays roles in regulating the transcription of the target genes by cooperating with other factors, such as p53^18^, the androgen receptor^19^, Smads^20^ and GATA2^21^. In our studies, PIASy was identified in an attempt to screen proteins interacting with Isl1 using the yeast two-hybrid system. Our results presented here demonstrate that PIASy is a novel factor that regulates insulin transcriptional activity and secretion by interacting with Isl1 in a synergistic manner and that the homeodomain of Isl1 and the ring domain of PIASy are required for the coactivation of the insulin promoter. These findings contribute to our understanding of the mechanism regulating insulin secretion.

## Results

### PIASy was Identified to Interact with Isl1 Using a Yeast Two-hybrid System

**Figure 1:**
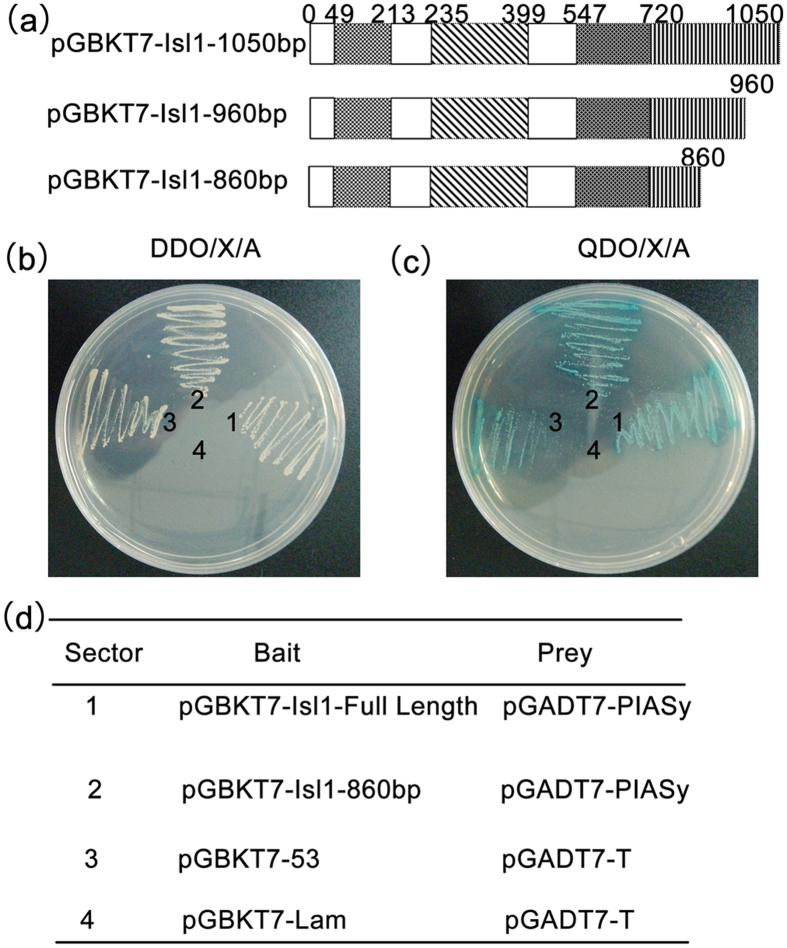
PIASy was identified as an Isl1 interacting protein with a yeast two-hybrid experiment. (**a**) Schematic of Isl1 baits used in the yeast two-hybrid experiment. (**b**) and (**c**) The bait plasmids and prey plasmids were transformed into Y2H gold yeast together as indicated in Fig. 1d, then the cultures were selected on DDO/X/A medium for three days incubation. Positive clones were transferred to a higher stringency QDO/X/A medium. The clones in sector 1 contained both the full length Isl1 and PIASy proteins. The clones in sector 2 contained both the deleted Isl1 and PIASy proteins. The clones in sector 3 and sector 4 contained positive and negative interaction controls, respectively, as the large T-antigen was known to interact with P53 but not with Lam in a yeast two-hybrid assay. (**d**) The designations in the table corresponded to four sectors on the plates in Fig. 1b and c. SD/-Leu/-Trp/X-a-Gal/AbA medium (DDO/X/A) means the medium supplemented with X-a-Gal and Aureobasidin A includes every essential amino acid except for leucine and tryptophan. SD/-Leu/-Trp/-His/-Ade/X-a-Gal/AbA medium (QDO/X/A) has the same components as DDO/X/A medium but also lacks histidine and adenine.

In our study, yeast two-hybrid system (Y2H) was used to identify proteins interacting with Isl1. For this screen, three different Isl1 mutations were constructed as Y2H baits and tested for autoactivation in the absence of prey proteins (Fig. 1a). Only the bait pGBKT7-Isl1-860bp did not autonomously activate the reporter genes in yeast and was used in conjunction with an adult testis cDNA library. Twenty-three positive clones were finally screened, three of which encoded PIASy. The interaction between full-length or deleted Isl1 and PIASy was further confirmed on DDO/X/A medium (Fig. 1b) and higher stringency QDO/X/A medium (Fig. 1c). These results indicated that PIASy is a novel Isl1-interacting protein.

### PIASy Interacts with Isl1 in Intact Cells

**Figure 2:**
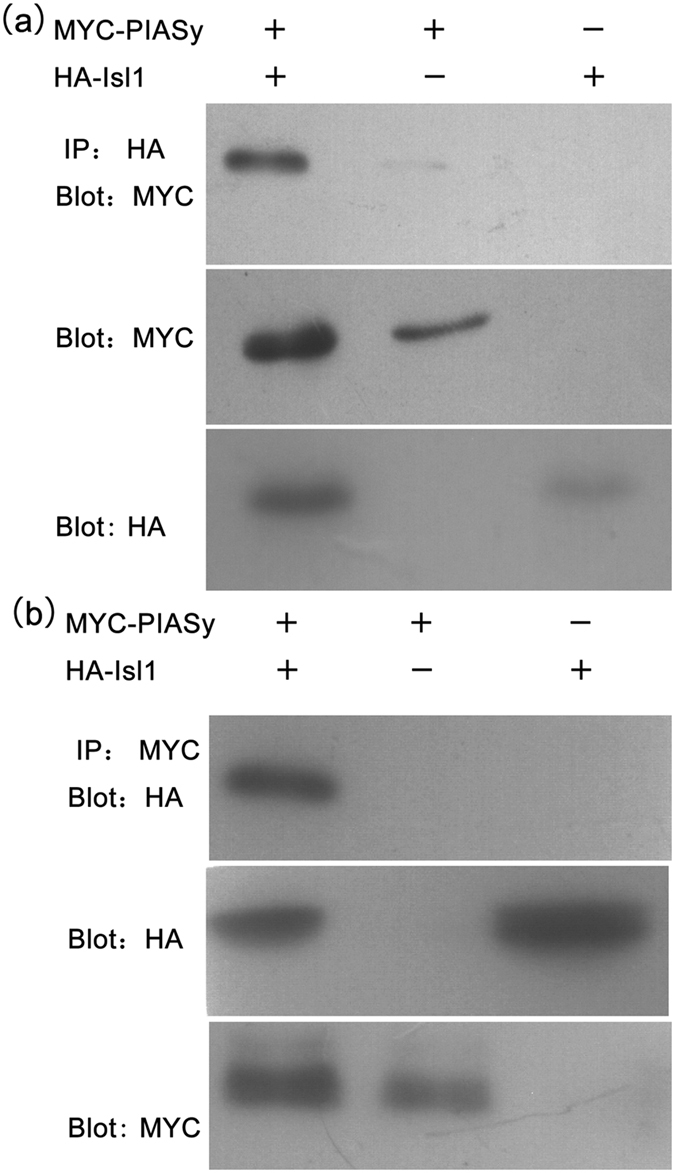
PIASy interacts with Isl1 in intact cells. (**a**) 293FT cells were transfected with the overexpression plasmids MYC-PIASy, HA-Isl1, or both. After culturing for 48 hours, cell lysates were harvested and incubated with anti-HA agarose followed by immunoblotting with an anti-MYC antibody, and MYC-tagged PIASy was detected. (**b**) Cell lysates were harvested and incubated with anti-MYC agarose followed by immunoblotting with an anti-HA antibody, and HA-tagged Isl1 was detected. In the bottom two panels, a MYC antibody or HA antibody was used to demonstrate the expression of tagged proteins.

To verify whether the interaction between the PIASy and Isl1 proteins observed in the Y2H screen also occurs in intact cells, a co-immunoprecipitation experiment was performed. Expression plasmids for HA epitope-tagged Isl1 and MYC epitope-tagged PIASy were transfected into 293FT cells, and then cell lysates were subjected to immunoprecipitation with an anti-HA antibody. The results of the immunoblotting analysis using the anti-MYC antibody showed that PIASy associated with HA-Isl1 but not with the vector control (Fig. 2a). The interaction between HA-Isl1 and MYC-PIASy was further demonstrated when cell lysates were subjected to immunoprecipitation with an anti-MYC antibody followed by immunoblotting with an HA antibody (Fig. 2b). These results confirmed the interaction between Isl1 and PIASy and suggested that Isl1 may form a complex with PIASy in intact cells.

### PIASy and Isl1 Colocalize in the Pancreas and NIT Cells

**Figure 3:**
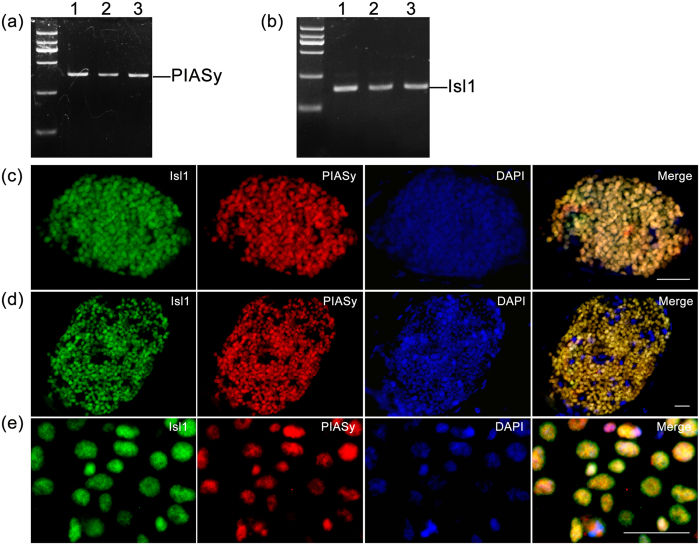
Isl1 and PIASy colocalize in mouse and human pancreas and NIT cells. (**a**) The gene expression of PIASy in mouse kidney (lane 1), the NIT cell line (lane 2), and mouse pancreatic islet (lane 3) as determined by common PCR is shown. (**b**) The gene expression of Isl1 in mouse kidney (lane 1), the NIT cell line (lane 2), and mouse pancreatic islet (lane 3) as determined by common PCR is shown. The kidney is positive for PIASy and Isl1 expression. (**c**) Dual staining of Isl1 (green) and PIASy (red) in the adult male mouse pancreas is shown. (**d**) Dual staining of Isl1 (green) and PIASy (red) in the human pancreas with pancreatic cancer is shown. (**e**) Dual staining of Isl1 (green) and PIASy (red) in the NIT cells is shown. Nuclei were stained with DAPI (blue). The merged images are on the right. Both Isl1 (green) and PIASy (red) showed a strictly nuclear localization and were coexpressed in most pancreas and NIT cells. Bars = 30 μm.

In our recent studies, Isl1 was suggested to regulate the effect of leptin and kisspeptin on insulin secretion in mice^22^,^23^. To identify whether the interaction between Isl1 and PIASy is involved in regulating insulin synthesis and secretion, first we detected the expressions of PIASy and Isl1 in mouse pancreatic islet and NIT beta cells using standard PCR and immunofluorescence. The NIT cell line is a pancreatic β cell line that is derived from mice with beta cell adenomas^24^. The PCR results indicated that PIASy (Fig. 3a) and Isl1 (Fig. 3b) were both detected in the pancreatic islets and NIT beta cells. The dual immunofluorescence results showed that Isl1 and PIASy were coexpressed in human and mouse pancreas (Fig. 3c and d). In addition, we tested Isl1 and PIASy coexpression in NIT cells (Fig. 3e). These results indicate the colocalization of Isl1 and PIASy in both the pancreas and NIT cells, and they are also in agreement with the above results that PIASy physically interacts with Isl1.

### PIASy Enhances the Effect of Isl1 on Insulin Gene Expression and Secretion

**Figure 4:**
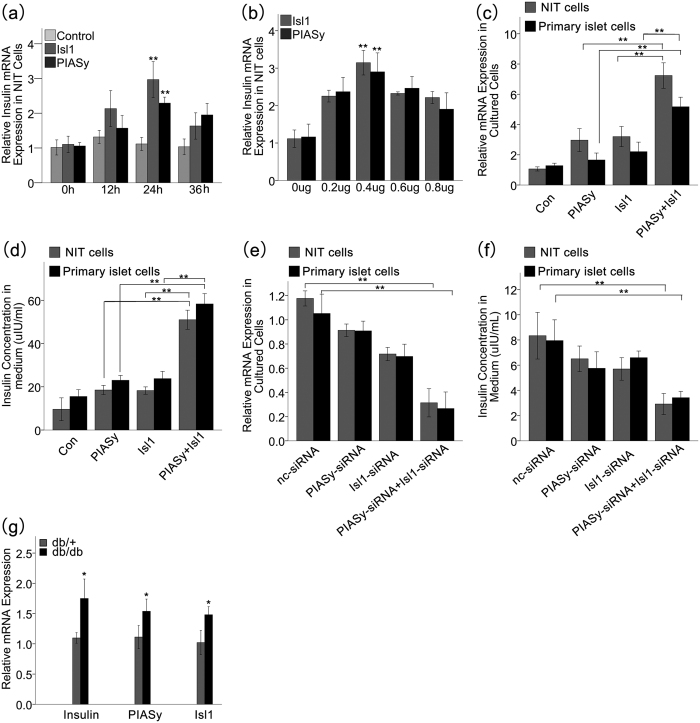
The interaction between Isl1 and PIASy protein promotes insulin gene expression and secretion. (**a**) Relative mRNA expression level of insulin in NIT cells transfected with Isl1 or PIASy overexpression vectors for various times was measured with real-time PCR. (**b**) Relative mRNA expression level of insulin in NIT cells transfected with different amount of Isl1 or PIASy overexpression vectors for 24 hours was measured with real-time PCR. (**c**) and (**d**) Shown are the relative insulin mRNA expression level and insulin concentration in NIT and primary pancreatic islet cells transfected with Isl1 and/or PIASy overexpression vectors at an amount of 0.4 μg for 24 hours. (**e**) and (**f**) NIT and primary pancreatic islet cells were transfected with PIASy-siRNA, Isl1-siRNA or nc-siRNA at an amount of 0.6 μg for 24 hours, and the relative insulin mRNA expression level and insulin concentration were detected. (**g**) Shown is the relative mRNA expression levels of insulin, PIASy and Isl1 in *db/db* and *db/*+ mouse islets (n = 3). The Insulin mRNA levels was quantified and normalized with that of GAPDH. Statistical analysis was completed using the Student’s t-test. Each bar is the mean ± SD from three samples. Significant differences are indicated by *(p < 0.05) and **(p < 0.01).

To assay the function of the interaction between Isl1 and PIASy on the transcription of insulin, we overexpressed the pCDNA3.1-Isl1 and pCDNA3.1-PIASy vectors in NIT cells. The results showed that both Isl1 and PIASy significantly enhanced the expression of insulin, which reached a peak 24 h after transfection (Fig. 4a). We then tested the effects of different amounts of the Isl1 and PIASy vectors on insulin expression, and the results showed that 0.4 μg of Isl1 or PIASy had the strongest effect at 24 h after transfections (Fig. 4b). Simultaneously, the insulin mRNA expression level and insulin concentration in cultured NIT and primary pancreatic islet cells were assayed. The enhancing effects of 0.4 μg Isl1 and PIASy cotransfection were much stronger than the single transfection of Isl1 or PIASy, although the insulin production in all of the constructs was higher than that in the control (Fig. 4c,d). Furthermore, 0.6 μg specific PIASy siRNA, Isl1 siRNA or negative control siRNA was transfected into NIT and primary pancreatic islet cells. The insulin mRNA expression level and insulin concentration decreased obviously when both PIASy siRNA and Isl1 siRNA were present (Fig. 4e,f). These findings demonstrate that PIASy mediates the effect of Isl1 on insulin gene expression and insulin secretion through proteins interactions. To study the possible effects of PIASy and Isl1 in physiological conditions, total RNA was extracted from the islets of *db/db* and *db/*+ mice and utilized for real–time PCR analysis. The mRNA levels of PIASy and Isl1 were increased in *db/db* mice relative to *db/*+ mice. This expression pattern of PIASy and Isl1 was consistent with that of insulin (Fig. 4g). These results suggest that PIASy and Isl1 may be related to hyperinsulinism and type 2 diabetes, but more details need to be elucidated.

### PIASy is a Transcriptional Cofactor of Isl1 that Regulates Insulin Promoter Activity

**Figure 5:**
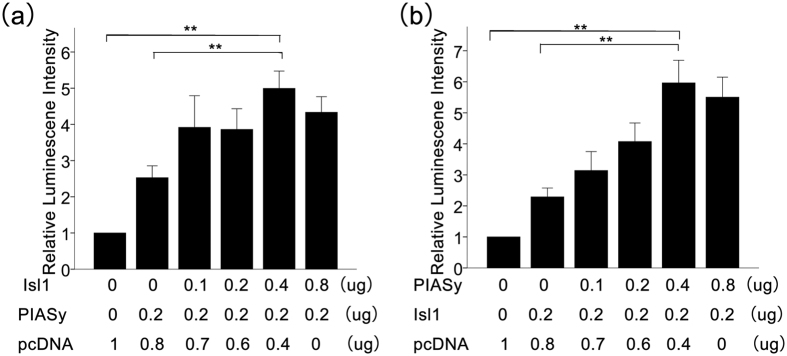
Isl1 and PIASy activate the insulin promoter in a synergistic manner. (**a**) Overexpression plasmids for 0.2 μg PIASy and different doses of Isl1 were transfected into NIT cells with luciferase reporter plasmids. The relative luminescence intensities were determined as described in the Materials and Methods. (**b**) Overexpression plasmids for 0.2 μg Isl1 and different doses of PIASy were transfected into NIT cells with luciferase reporter plasmids. The empty vector pcDNA3.1 was used to normalize the transfection efficiency. Relative luminescence intensities were determined as described in the Materials and Methods. Statistical analysis was completed using the Student’s t-test. Each bar is the mean ± SD from three independent experiments. Significant differences are indicated by *(p < 0.05) and **(p < 0.01).

Since PIASy and Isl1 have synergistic effects in elevating insulin mRNA expression and secretion, we identified whether PIASy was involved in regulating insulin promoter activity with Isl1. A firefly luciferase reporter plasmid pGL3-Insulin containing the mouse insulin promoter (0 to −500 bp) and a control Renilla luciferase reporter plasmid were cotransfected into NIT cells with the pCDNA3.1-Isl1 and/or pCDNA3.1-PIASy plasmids. As shown in Fig. 5a,b, Isl1 and PIASy together activated the insulin promoter more than either Isl1 or PIASy alone. In addition, greater amounts of the PIASy or Isl1 plasmids had a more significant influence. These data suggest that PIASy and Isl1 mediate insulin expression and secretion by activating the insulin promoter in a synergistic manner.

### The Homeodomain of Isl1 and Ring Domain of PIASy are Responsible for the Coactivation of the Insulin Promoter

**Figure 6:**
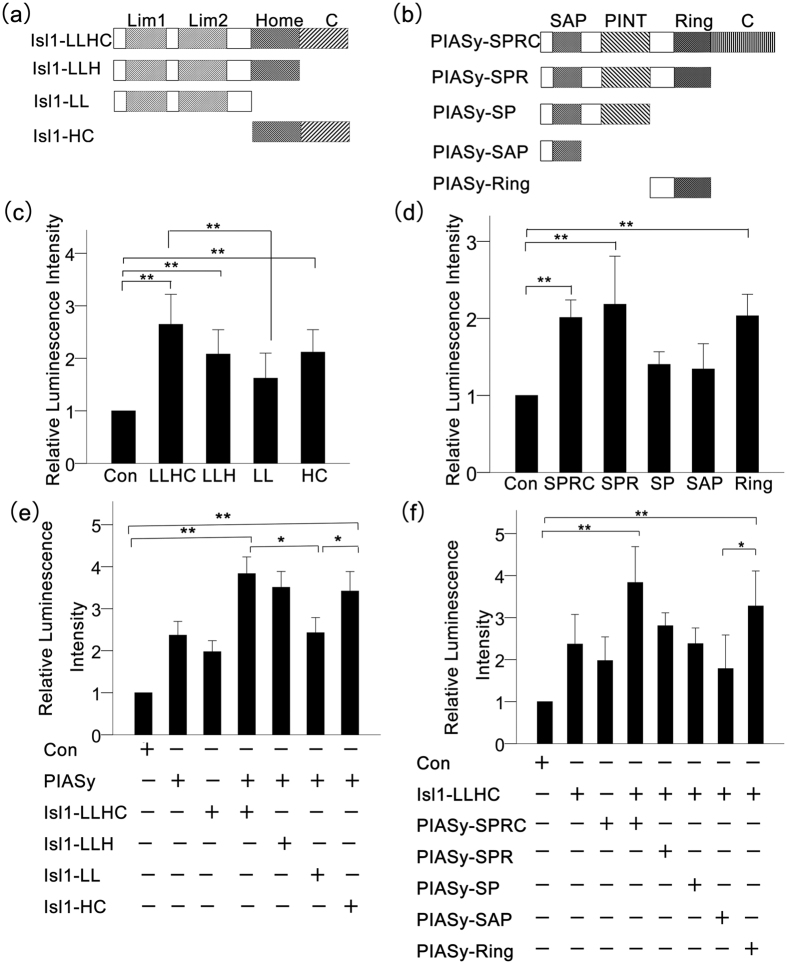
The homeodomain of Isl1 and ring domain of PIASy are responsible for coactivation of the insulin promoter. (**a**) A schematic illustration of mutated Isl1 constructs. (**b**) A schematic illustration of mutated PIASy constructs. The different vectors are named after the domains of Isl1 or PIASy shown above the scheme of the full length protein structure. (**c**) and (**d**) Isl1 or PIASy mutants (0.4 μg) were transfected into NIT cells as indicated, and then the relative luminescence activities were tested as described. (**e**) Mapping the full-length PIASy with various Isl1 domains for the coactivation of the insulin promoter. Isl1 and/or PIASy mutants (0.4 μg) were transfected into NIT cells as indicated. (**f**) Mapping the full length Isl1 with various PIASy domains for the coactivation of the insulin promoter. Isl1 and/or PIASy mutants (0.4 μg) were transfected into NIT cells as indicated. Statistical analysis was completed with the Student’s t-test. Each bar is the mean ± SD from three independent experiments. Significant differences are indicated by *(p < 0.05) and **(p < 0.01).

To further map the domains of Isl1 and PIASy required for insulin promoter coactivation, different Isl1 and PIASy deletions were constructed (Fig. 6a,b), and 0.4 μg of the Isl1 or PIASy mutants were transfected into NIT cells. Compared with the full-length Isl1 (Isl1-LLHC), insulin promoter activity was significantly lower in the Isl1-LL mutant, which lacked both the homeodomain and the C-terminal domain (Fig. 6c). The activity of the insulin promoter was also obviously reduced after the cotransfection of the full-length PIASy (PIASy-SPRC) and Isl1-LL, but not Isl1-LLHC, Isl1-LLH or Isl1-HC (Fig. 6e). All three of these constructs had a common homeodomain structure. These results demonstrate that the homeodomain of Isl1 is important for the activation of the insulin promoter.

As shown in Fig. 6d, the deletion of the C-terminal domain (PIASy-SPR) had no effects on the function of PIASy, and the PIASy-SP and PIASy-SAP mutants, both of which had a common feature in lacking of the ring domain, failed to activate the insulin promoter. Interestingly, the ring domain (PIASy-Ring) alone restored the activation. To further research the domains of PIASy responsible for coactivation of the insulin promoter, we cotransfected Isl1-LLHC and the indicated truncated PIASy mutants into NIT cells, and found that the function of PIASy-Ring was consistent with the above results (Fig. 6f). These collective data suggest that both the Isl1 homeodomain and PIASy ring domain play important roles in the coactivation of the insulin promoter.

## Discussion

Isl1 is known as a LIM homeodomain transcription factor, and it regulates target genes expression by interacting with various specific transcriptional factors and coregulators^25^,^26^. A method to screen for Isl1-interacting proteins on a large scale has not been established. In this study, we identified PIASy as a novel Isl1-interacting protein using a yeast two-hybrid system and revealed a new role of PIASy as a cofactor of Isl1 in the regulation of insulin secretion. In support of this finding, PIASy and Isl1 colocalize in the nuclei of pancreas and NIT beta cells. Furthermore, we demonstrated that PIASy promotes the effect of Isl1 on insulin gene expression and secretion by activating the insulin promoter. The results of the present study thus provide us with new insight into the role of protein interactions in regulating insulin expression and secretion, although more details will be determined in future studies.

Protein-protein interactions are essential for biological processes and lead to the new ideas about protein functions^27^,^28^, so identifying novel proteins that interact with Isl1 helps to elucidate the mechanism by which Isl1 regulates insulin synthesis and secretion. To screen for Isl1-interacting proteins using a yeast two-hybrid system, we used the truncated Isl1 mutant as a new bait due to the autoactivation of the full-length Isl1, and PIASy was identified as a novel Isl1 interacting protein. Their interaction was subsequently confirmed with a co-immunoprecipitation experiment. Both Isl1 and PIASy have been found to interact with many coregulators. For example, Isl1 associates with Ldb1 through its C-terminal domain, and this interaction is critical in the specification of motor neuron identity^29^,^30^. In the rat hypothalamus, Isl1 interacts with the oestrogen receptor (ER) and prevents dimerization of ER, which specifically decreases ER binding to DNA and transcriptional activation activities^31^. PIASy interacts with the androgen receptor (AR) and represses its transcription without inhibiting the ability of AR to bind to DNA sequences. However, PIAS1 and PIAS3 activate the transcription of AR compared with PIASy, although they all have an RD1-related domain, which is required for the regulation of AR^19^,^32^. In addition, PIASy also interacts with E12, p73 and TBP^33^,^34^,^35^. Isl1 and other LIM proteins require cofactors to effectively bind to DNA sequences and regulate target gene transcription, and PIASy may play that role as a new cofactor of Isl1.

As a key transcription factor, Isl1 plays a significant role in pancreas. Isl1 not only is required for the formation of pancreatic mesenchyme and islet cells but also regulates the secretion of endocrine hormones such as somatostatin^36^, proglucagon^37^ and insulin^11^ in the pancreatic islets. Complex cell signalling pathways intimately regulate insulin expression in the pancreatic cells. Our recent studies suggest that Isl1 regulates the effect of leptin and kisspeptin on insulin secretion in mice^22^,^23^, so we speculate that the interaction between PIASy and Isl1 may be involved in mediating insulin gene expression and secretion. Coinciding with the above thought, both PIASy and Isl1 are expressed in human and mouse pancreas and NIT beta cells. By transfecting PIASy and Isl1 expression vectors or inhibitor vectors into primary islet cells and NIT beta cells, we found that PIASy enhances the effect of Isl1 on insulin gene expression and secretion. PIASy and Isl1 mRNA expression levels were also increased in *db/db* mice with hyperglycaemia, which have been used as a type 2 diabetes model^38^. The higher expression of PIASy and Isl1 may be a cause of hyperinsulinemia. In support of this, Isl1 mutations have been found in French and Japanese patients with type 2 diabetes^14^,^15^. These findings suggest that PIASy and Isl1 synergistically regulate insulin gene expression and secretion through protein-protein interactions and may be candidate genes for type 2 diabetes treatments, in addition to providing a new view about the pathogenesis of type 2 diabetes.

Isl1 regulates the transcription of target genes by binding to its recognition sites in the promoter region in cooperation with other cofactors. It has been reported that Isl1 activates insulin gene transcription with BETA2 by activating the insulin promoter in the pancreas^39^. In pancreatic β cells, Isl1 could activate the rat insulin promoter by binding to the highly conserved A3/4 box of the insulin promoter^40^,^41^. The combination of Isl1 and GATA4 could bind to the promoters of Mef2c and Nkx2.5 in a similar manner^42^. Gene transcription is usually regulated by multiple interactions. In our study, the interaction between Isl1 and PIASy was found to significantly enhanced insulin promoter activity in a synergistic manner. In mapping the Isl1 and PIASy domains responsible for coactivation of the insulin promoter, Isl1 constructs lacking a homeodomain and PIASy constructs lacking a ring domain decreased insulin promoter activity; thus, the homeodomain and ring domain are required for insulin promoter coactivation. In other studies, the LIM domain of Isl1 was shown to be responsible for proteins interactions and the homeodomain of Isl1 directly binds to DNA sequences^43^,^44^; thus, the loss of function in Isl1-LL may be attributed to the inability to bind to DNA. The ring domain is usually regarded as the most conserved domain of the PIAS family, and it is also responsible for proteins interactions^45^. Based on these data, we speculate that Isl1 exposes its homeodomain and binds to the insulin promoter by interacting with the ring domain of PIASy, but more details need to be analysed at the molecular level.

Taken together, the present study suggests that PIASy is a novel factor that regulates insulin gene expression and secretion through its interaction with the LIM-HD transcription factor Isl1. Both PIASy and Isl1 colocalize in the pancreas and NIT beta cells, and PIASy enhances the effect of Isl1 on insulin gene expression and production by activating the insulin promoter in a synergistic manner. We also indicate that the homeodomain of Isl1 and the ring domain of PIASy are critical for the coactivation of the insulin promoter. These results offer new insight into the mechanism underlying insulin expression and secretion and are helpful in better understanding the pathogenesis of type 2 diabetes.

## Materials and Methods

### Animals

Adult (8-week-old) male C57BL/6 mice and male *db/db* mice were used in this study. All animal studies were approved by the Chinese Association for Laboratory Animal Sciences. The studies were carried out in accordance with the relevant guidelines, including any relevant details. The *db/db* mice were used as the type 2 diabetes animal model, and *db*/+ mice were used as the control group^38^. The *db/db* mice from The Jackson Laboratory were 12 weeks old and showed a diabetic phenotype. Total RNA was extracted from the pancreatic islets and utilized for real–time PCR analysis.

### Patient material

One patient with pancreatic cancer was selected from the 306th Hospital of the People’s Liberation Army, Beijing. The pancreatic tissue was from excess material collected from the patient, who was undergoing surgery to retrieve surgical specimens. Written informed consent was obtained from the patient. This study was approved by instructions and guidelines of the 306th Hospital Ethics Committee and Chinese Association for Laboratory Animal Sciences. The methods were carried out in accordance with the relevant guidelines, including any relevant details.

### Yeast Two-Hybrid Screening

Full-length Isl1 and the truncated mutation lacking 40 amino acids at the C-terminus were amplified with primers containing a 5′ *NdeI* site and a 3′ *XhoI* restriction enzyme site using cDNA as a template; these constructs were then cloned into the bait pGBKT7 vectors (BD Biosciences Clontech, CA). The plasmids were transformed into the *Saccharomyces cerevisiae* strain Yeast Gold and were tested for autoactivation and toxicity. Subsequently, the bait pGBKT7-Isl1–860bp vectors were transformed into Yeast Gold, which was then mated with yeast containing a mouse testis cDNA library. The yeast were plated onto 150 mm plates containing SD/-Leu/-Trp/X-a-Gal/AbA (DDO/X/A) and incubated for 5 days at 30 °C, and then the resulting clones were transferred onto SD/-Leu/-Trp/-His/-Ade/X-a-Gal/AbA (QDO/X/A). The plasmids were obtained from the final positive clones and amplified by transformation into DH5α competent cells. The single bait and prey plasmids were cotransformed into Yeast Gold after sequencing, and then the yeast clones were patched onto DDO/X/A and QDO/X/A medium for confirmation.

### Co-immunoprecipitation

A co-immunoprecipitation experiment was completed to ascertain the interaction between Isl1 and PIASy. The pXJ40-HA-Isl1 expression vector was a kind gift from Dr. Xinmin Cao (Institute of Molecular and Cell Biology, Singapore). The prey plasmid pGADT7-PIASy was used as a template in polymerase chain reaction (PCR) to amplify the full length PIASy. The fragments were digested with *BamHI* and *HindIII* endonucleases and inserted into the pXJ40-MYC–tagged plasmid for overexpression in mammalian cells. The 293FT cells were transfected with pXJ40-HA-Isl1 and/or pXJ40-MYC-PIASy plasmids. The cells were harvested after 48 hours and lysed in nondenaturing lysis buffer. The cell lysate was incubated with anti-HA (26180, Thermo Scientific, Rockford, IL, USA) or anti-MYC agarose beads (23620, Thermo Scientific, Rockford, IL, USA) at 4 °C overnight. The eluted proteins were blotted with anti-MYC or anti-HA antibodies at a dilution of 1:1000 (Santa Cruz Biotechnology, USA).

### Double Staining Immunohistochemistry

Pancreatic samples were first fixed in freshly prepared 4% paraformaldehyde and embedded in paraffin. Sections were dewaxed, rehydrated, and blocked in 10% normal donkey serum for 2 h. Sections were then incubated with an anti-Isl1 antibody diluted 1:150 (AF1837; R&D, USA) at 4 °C overnight before being rinsed three times and incubated with a donkey anti-goat antibody diluted 1:200 (DAR-488, Life Technologies, USA) for 3 hours at room temperature. After three rinses and overnight incubation with an anti-PIASy antibody diluted 1:200 (sc-166706, Santa Cruz Biotechnology, USA) at 4 °C, the sections were rinsed three times and incubated with a donkey anti-mouse antibody diluted 1:200 (DAM-555, Life Technologies, USA) for 3 hours at room temperature. After three final rinses, the sections were examined under a fluorescence microscope (Leica Microsystems, Cambridge, UK). The NIT cell immunofluorescence assay was performed as previously described^7^.

### Cell Culture and Transfection

Pancreatic islets were isolated and cultured as described previously^22^,^46^. The NIT pancreatic β cell line and human embryonic kidney 293FT cells were maintained in Dulbecco’s Modified Eagle Medium (DMEM) with 10% foetal bovine serum and 1% penicillin-streptomycin at 37 °C with 5% CO_2_. PIASy siRNA, Isl1 siRNA and NC siRNA were constructed by Gene Pharma Company (Shanghai, China). The Isl1 siRNA sequences were: sense, GGACCAGGCUCUAAUUCUATT; antisense, UAGAAUUAGAGCCUGGUCCTT. The PIASy siRNA sequences were: sense, GAUGAGCUUCCGAGUAUCATT; antisense, UGAUACUCGGAAGCUCAUCTT. The stable negative control siRNA sequences were: sense, UUCUCCGAACGUGUCACGUTT; antisense, ACGUGACACGUUCGGAGAATT. All transient transfections were performed using Lipofectamine 2000 (Invitrogen) according to the manufacturer’s recommendations.

### Real-time PCR (RT-PCR)

Total RNA was extracted from cultured cells and cDNA was generated from 2 μg RNA in a 25 μl reaction mixture using M-MLV reverse transcription reagents (M170A; Promega, USA). Real-time PCR amplification was performed three times (DRR420A; Takara, Dalian, China) in the ABI PRISM 7500 sequence detection system (Applied Biosystems, Foster City, USA). The insulin primers used for RT-PCR were as follows: sense 5′-AGGACCCACAAGTGGAACAACT-3′; antisense 5′-CAACGCCAAGGTCTGAAGGT-3′. The Isl1 primers were as follows: sense 5′-CTGCTTTTCAGCAACTGGTCA-3′; antisense 5′-AGGACTGGCTACCATGCTGT-3′. The PIASy primers were as follows: sense 5′-TCCGCAGGAGGACCAATACC-3′; antisense 5′-CAGGTGACAGTAATGCGGTTGG-3′. The GAPDH primers were as follows: sense 5′-GGTTGTCTCCTGCGACTTCA-3′, antisense 5′-GGGTGGTCCAGGGTTTCTTA-3′. For normalization purposes, an identical set of reactions was prepared for GAPDH.

### Radioimmunoassays (RIA)

Radioimmunoassays were performed as described previously using the insulin radioimmunoassay reagents provided by the Beijing North Institute Biological Technology (Beijing, China)^22^,^23^. For each radioimmunoassay the intra-assay and inter-assay coefficients of variation were less than 10% and 15%.

### Plasmids Constructs and Dual Luciferase Assays

The fragment containing the insulin promoter (0 to −500 bp) was amplified from mouse genomic DNA using the PCR method and inserted into the pGL3 vector (E1910, Promega). All the Isl1 and PIASy mutant fragments were amplified with the PCR method using the primers containing the *NheI-XhoI* restriction sites as shown in Table 1 and inserted into the pcDNA3.1 vector. Different Isl1 and PIASy vectors were transfected into NIT cells with the luciferase reporter vector pGL3-Insulin and the pTK-Renilla vector, and the empty vector pcDNA3.1 was used to normalize the transfection efficiency. The cells were harvested 48 h after transfection, and luciferase and Renilla activities were measured using a dual luciferase assay kit (Vigorous, Beijing, China).

### Statistical analysis

All data were expressed as the means ± SD. Statistical analysis was performed with SPSS 20.0 (SPSS Inc., Illinois, USA). Comparisons between two groups were analysed using the Student’s *t*-test. A value of *p *< 0.05 was considered to be statistically significant.

## Additional Information

**How to cite this article**: Yan, C. *et al*. Protein Inhibitor of Activated STAT Y (PIASy) Regulates Insulin Secretion by Interacting with LIM Homeodomain Transcription Factor Isl1. *Sci. Rep.* **6**, 39308; doi: 10.1038/srep39308 (2016).

**Publisher's note:** Springer Nature remains neutral with regard to jurisdictional claims in published maps and institutional affiliations.

## Figures and Tables

**Table 1:** The PCR primer sequences used for plasmid construction.

Gene	Primer sequence (5′–3′)
Insulin Promoter	Sense 5′-GCCTGAGTTCTGCTTTCC-3′
	Antisense 5′-CTGCTTGCTGATGGTCTCTGATTAT-3′
Isl1-LLHC	Sense 5′-CTAGCTAGCATGGGAGACATGGGCGAT-3′
	Antisense 5′-CCGCTCGAGTCATGCCTCAATAGGACTGG-3′
Isl1-LLH	Sense 5′-CTAGCTAGCATGGGAGACATGGGCGAT-3′
	Antisense 5′-CCGCTCGAGGCTGCGTTTCTTGTCCTT-3′
Isl1-LL	Sense 5′-CTAGCTAGCATGGGAGACATGGGCGAT-3′
	Antisense 5′-CCGCTCGAGGTGGTCTGCACGGCAG-3′
Isl1-HC	Sense 5′-CTAGCTAGCGATGTGGTGGAGAGAGCCAG-3′
	Antisense 5′-CCGCTCGAGTCATGCCTCAATAGGACTGG-3′
PIASy-SPRC	Sense 5′-CTAGCTAGCATGGCGGCAGAGCTGGTGG-3′
	Antisense 5′-CCGCTCGAGTCAGCACGCGGGCACCAGGCCT-3′
PIASy-SPR	Sense 5′-CTAGCTAGCATGGCGGCAGAGCTGGTGG-3′
	Antisense 5′-CCGCTCGAGAGGCTTGTCACACACAGGGC-3′
PIASy-SP	Sense 5′-CTAGCTAGCATGGCGGCAGAGCTGGTGG-3′
	Antisense 5′-CCGCTCGAGTCGCACCAGGTACAAAGCCACAG-3′
PIASy-SAP	Sense 5′-CTAGCTAGCATGGCGGCAGAGCTGGTGG-3′
	Antisense 5′-CCGCTCGAGCACCAGCTGCAAGGCCCTGGTC-3′
PIASy-Ring	Sense 5′-CTAGCTAGCCAGCTGACCTCCTCAGACCT-3′
	Antisense 5′-CCGCTCGAGAGGCTTGTCACACACAGGGC-3′
